# Mobile Phone Addiction and Its Relationship to Sleep Quality and Academic Achievement of Medical Students at King Abdulaziz University, Jeddah, Saudi Arabia

**Published:** 2018-08-04

**Authors:** Nahla Khamis Ibrahim, Bashaer Saleh Baharoon, Waad Fouad Banjar, Anfal Abdulrahman Jar, Roba Mahmod Ashor, Alanoud Akram Aman, Jawaher Rabah Al-Ahmadi

**Affiliations:** ^1^ Family and Community Medicine Department, Faculty of Medicine, King Abdulaziz University, Jeddah, Saudi Arabia; ^2^ Department of Epidemiology, High Institute of Public Health, Alexandria University, Alexandria, Egypt; ^3^ Interns, Faculty of Medicine, King Abdulaziz University, Jeddah, Saudi Arabia

**Keywords:** Mobile phone, Dependency, Sleep, Academic performance, Medical students

## Abstract

**Background:** Adverse effects of Mobile Phone (MP) usage could lead to dependency problems, and medical students are not excluded from it. We aimed to determine the pattern of MP usage, and its relation to sleep quality and academic performance between medical students at King Abdulaziz University (KAU), Jeddah, Saudi Arabia.

**Study design:** A cross-sectional study.

**Methods:** A multistage stratified random sample was used for selection of 610 participants, during 2016- 2017. A validated, anonymous data collection sheet was used. It inquired about the Grade Point Averages (GPA). It included the Problematic Mobile Phone Use Questionnaire (PMPU-Q) for assessing various aspects of cellphone addiction (dependency, financial problems, prohibited and dangerous use). The Pittsburgh Sleep Quality Index (PSQI) was also included. Descriptive and inferential statistics were done.

**Results:** A high frequency of MP usage prevailed among participants (73.4% used it >5 h/day). About two-thirds of participants had poor sleep quality. Females, owners of smartphone for >1 yr, and increasing time spent on MP were associated with MP dependency. Lower academic achievers had significantly worse MP scores on financial problems, dangerous use, and total PUMP. MP dependency was correlated with subjective sleep quality score, and sleep latency. Global PSQI scale was correlated with prohibited MP use.

**Conclusions:** Lower achievers had significantly worse scores on MP financial problems, dangerous usage, and the total PMPU. MP dependency was correlated with poor subjective sleep quality, and sleep latency. Rationale MP usage is needed to decrease the dependency, improve sleep quality, and academic achievement of medical students.

## Introduction

Under the extensive technological revolution, the mobile phone (MP) usage has rapidly increased^1^. Nowadays, smartphone is not just used for phone-calls and text-messaging, but it goes beyond that. It reached out to include internet access to multimedia through social networks, video games and Global Positioning System (GPS) navigation^1,2^. MPs are now utilized globally as one of the chief information and communication technologies (ICTs)^1^. The smartphones have also proved to be useful for medical students as a potential tool to “learn anywhere”^3^.

Despite the benefits, there are many adverse effects of the smartphone irrational usage. MP could lead also to dependency problems^4-6^ characterized by excessive and continuity of performing an activity despite its negative outcomes. This includes mental stress, feeling of being captivated, role conflicts, and obligatory feelings to respond to all notifications, calls, and messages.Nomo-phobia is a fear of not having the MP around. Saudi Arabia ranked the first of all countries of Gulf Cooperation Council regarding the proportion of MP users^4^.

Medical students are not excluded from MP addiction; due to more MP engagement in their daily life^7^. Furthermore, sleep is crucial for preserving the person’s physical and mental health. Smartphone usage might lead to sleep disturbance^8^, which may affect the concentration level and academic performance^9^. However, few types of research were done to determine the pattern of MP usage, and its relationship of usage with sleep quality and academic achievements^10^,especially among medical students in Jeddah.

We aimed to determine the pattern of MP usage, and its relation to sleep quality and the academic performance of medical students at King Abdulaziz University (KAU).

## Methods

This cross-sectional study was conducted during the academic year 2016‏-2017. All medical students who completed the freshman year (from the 2^nd^-6^th^ year), and accepted to participate in the study were eligible to enroll. A multistage stratified random sample method was used to select medical students. Students’ gender and educational level were considered for the stratification purpose. The sample size was calculated by the formula of calculation of sample from the cross-sectional study. Since the rate of problematic MP usage between medical students in Jeddah, other parts of Saudi Arabia by the used tool is unknown, so “*P*” was supposed to equal “q” (0.5) as the most conservative sample in this case^11^. The minimum calculated sample size for achieving a precision of ± 4%, at 95% confidence interval (C.I.) was 600 students.

A validated, confidential, anonymous data collection sheet was used. It‏ inquired about personal and socio-demographic data. It assessed also the Grade Point Averages (GPA) during last semester. The questionnaire included the English version of Pittsburgh Sleep Quality Index (PSQI)^12^. The English version has a good validity and reliability^13,14^. The data sheet contained also the standardized English version of the Problematic Mobile Phone Use-Questionnaire (PMPU-Q)^15,16^. This tool comprises thirty items for assessment of various aspects of cellphone addiction (dependency, financial problems, prohibited & dangerous use). PMPU-Q contains also general questions about MP and Short Message Service (SMS) usage. It has good validity and reliability. Its internal consistency reliability was assessed by Cronbach's alpha and found to range from 0.85 to 0.90^15^. For the dangerous MP domain, a mild modification was done to fit for females in Saudi Arabia and re-validation was done.

### Statistical analysis

SPSS ver. 21 (Chicago, IL, USA) was used. The PMPU-Q score was calculated with its four domains^10,14^. Nineteen self-rated PSQI were combined into seven components^12^. Descriptive statistics using proportions, means and standard deviations (SDs) was done. Inferential statistics were conducted. Student's *t*-test and ANOVA were used for comparing between means. Post-hoc Least Statistical Difference (LSD) was calculated. Pearson's correlation coefficient (r) was used to measure the strength of the association between two continuous variables

### Ethical statement

The research was confirmed to the ethical standards of Helsinki declaration. The Institutional Review Board (IRB) of KAU approved the study with a Reference Number: 92-16. Each student accepted to participate in the study signed a written informed consent.

## Results

The study enrolled 610 participates; with a slight increase than minimal sample for the stratification purpose. The male: female ratio was 1.06:1. The mean (± SD) of the age was 21.60± 2 yr. According to PSQI, 68.4% of the students had poor sleep quality, and the actual sleeping hours per day was 6.0 ± 1.84 h.

All participants had MPs, and 73.4% of them used their MP for >5 h per day (average of 3.0 h). The most frequently used mobile's applications were What’s App (45%), followed by Snap Chat (35%), YouTube (25%), Instagram (19%), Twitter (19%), Path (11%) and Facebook (7%). The mean checking What’s App was 10 times per day.

Table 1 shows that older students (aged ≥22 yr), and those enrolled in the clinical years had significantly (*P*<0.001) worse scores regarding the financial problems, dangerous MP use and the total PUMP score compared to others. Furthermore, females had more MP dependency. The mean MP dependency score was significantly higher among females compared to males (*P*<0.001). On the other hand, males had worse scales on the financial problems, dangerous mobile use, and the total PUMP compared to females. Students who owned smartphones for more than 5 yr, and those spent>30 min/day on their MP obtained significantly higher scores in the dependency, dangerous mobile use, and total PUMP compared to others. Regarding the relationship between MP use and the academic achievement, the table shows that students with lower GPA had significantly worse scores on MP financial problems, dangerous MP usage, and the total PMPU compared to their counterparts with higher scholastic achievements.‏ Those who reported an increased frequency of calls and SMS had increased MP financial problems, dangerous MP use and total PMPU score than others. However, there is no relationship between MP use and income.

**Table 1:** Relationship between domains of problematic mobile phone usage and the characteristics of medical students at King Abdulaziz University, Jeddah, Saudi Arabia

**Variables**	**MP** **Dependence**		**Financial** **problems**		**Prohibited** **use**		**Dangerous** **use**		**Total** **PUMP Score**	
	**mean**	**SD**	**mean**	**SD**	**mean**	**SD**	**mean**	**SD**	**mean**	**SD**
Age (yr)										
<22	19.07	3.74	27.84	5.38	11.01	2.29	11.63	2.76	69.83	9.08
≥22	13.75	2.75	31.12	6.39	10.84	2.46	13.57	2.75	74.40	10.55
*P* value	0.097		0.001		0.388		0.001		0.001	
Gender										
Male	18.28	3.14	31.41	6.43	11.00	2.37	12.69	2.91	73.20	10.32
Female	19.38	4.11	27.32	5.01	10.18	1.65	10.00	2.20	67.00	9.77
*P* value	0.001		0.001		0.378		0.001		0.001	
Academic year										
Basic	18.77	3.74	27.79	5.54	11.00	2.21	11.29	2.78	68.99	9.75
Clinical	18.84	3.64	30.51	6.27	10.87	2.48	13.45	2.74	74.20	10.38
*P* value	0.846		0.001		0.505		0.001		0.001	
Grade Point										
<4.5	18.67	3.38	30.11	6.46	10.87	2.39	13.15	2.84	73.54	10.55
≥4.5	19.09	3.61	28.53	5.42	10.86	2.23	11.83	2.99	70.44	9.84
*P* value	0.192		0.005		0.948		0.001		0.010	
Income (SR/month)										
≥10,000	18.85	3.69	29.37	6.10	10.89	2.36	12.62	2.95	73.34	10.35
<10,000	17.65	3.37	30.71	6.56	11.88	2.96	12.08	2.84	72.36	12.41
*P* value	0.185		0.375		0.090		0.679		0.940	
Duration of having a smartphone (yr)								
<1	11.5	6.36	25.50	13.44	11.00	4.24	9.50	2.12	57.50	26.16
1‏-5	18.5	3.67	28.88	5.66	11.09	2.32	12.01	2.86	70.19	9.69
>5	18.97	3.65	29.63	6.19	10.88	2.39	12.92	2.89	73.18	10.46
*P* value	0.007		0.277		0.616		0.014		0.008	
Time spent on mobile phone per day (min)							
0-10	15.69	5.59	28.54	6.85	10.31	3.17	10.11	3.10	61.44	17.69
>10	18.83	3.62	29.46	6.13	10.91	2.37	12.23	3.12	73.69	9.61
*P* value	0.002		0.590		0.368		0.040		0.001	
Calls made per day										
0-2	18.84	3.85	27.27	5.50	10.79	2.29	11.35	2.81	67.74	10.20
3-5	18.60	3.55	30.53	5.89	11.10	2.47	12.61	2.92	74.08	9.72
>5	19.31	3.57	32.69	5.34	10.87	2.37	13.44	2.82	76.85	9.66
*P* value	0.294		0.001		0.308		0.001		0.001	
SMS sent per day										
0-3	18.87	3.81	28.80	5.96	10.77	2.39	12.32	2.88	71.19	10.17
4-10	18.19	3.64	29.18	6.41	11.22	2.49	13.22	2.69	73.64	12.01
>10	19.17	3.19	31.26	6.04	11.18	2.26	13.27	2.93	74.62	10.07
*P* value	0.144		0.001		0.120		0.030		0.030	

Table 2 shows that there are significant correlations between MP dependency with each of subjective sleep quality (*P*<0.01), and sleep latency (*P*<0.05) domains of the PSQI. Furthermore, prohibited MP scale was significantly correlated with subjective sleep quality, sleep latency, sleep disturbance, use of sleep medication and the total PSQI scale. Furthermore, dangerous MP use was significantly correlated with sleep latency and the usage of sleep medication. Academic achievement was negatively correlated with dangerous mobile use and financial problems.

**Table 2:** Correlation coefficients between sleep quality components, academic achievement and the domains of Problematic Mobile Phone among medical students at King Abdulaziz University, Jeddah, Saudi Arabia

**PSQI Components**	**Prohibited use**	**Financial problems**	**Dangerous use**	**Dependency**
Subjective sleep quality score	0.100	-0.046	-0.070	0.136
Sleep latency	0.100	-0.070	-0.142	0.100
Sleep duration	0.053	-0.026	-0.042	0.034
Sleep efficiency	-0.022	-0.030	0.006	0.049
Sleep disturbance	0.153	0.090	-0.006	-0.058
Use of sleep medication	0.087	0.223	0.146	-0.062
Daytime dysfunction	0.008	-0.062	-0.060	0.058
Total PSQI score	0.112	0.004	-0.042	0.069
Academic achievement	0.026	-0.127	-0.138	0.036

## Discussion

In the current study, females were significantly more dependent on MP than were males. This result supports the preceding studies from Turkey^2^ and Korea^17^. These findings might be because females usually like the talk and to do interpersonal interaction with her relatives and friends more than males^17^. On the other hand, absence of such association was reported from another Turkish study ^8^. In the current study, males had worse scales on financial problems, dangerous MP usage, and with the total PUMP scales compared to females. The reason for that might be due to the differences between social engagements among both genders in Saudi culture.

A study done by college students from the USA demonstrated that MP usage was negatively associated with their GPA^18^, which is in line with our findingd. These results are also consistent with other previous studies^19,20^. Such findings may be because modern MPs create accessing to Internet, checking social media, playing video games, contacting friends, exploring new applications, or engaging with many MP leisure activities. These can lead to decrease the concentration levels and sleep quality, and cause wasting of more time on MP. Such time is needed for more studying and obtaining better GPA.

More than two-thirds of our participants had poor sleep quality (PSQI), which agrees with the previous studies from Palestine^21^ and Jeddah^22^. In San Francisco, California, the longer average spent on the screen-times through the time on bed was associated with poor quality of sleep, diminished sleep efficiency, and increased in the sleep onset latency.^23^ Our results were consistent with the results from California as MP dependency score was associated with the subjective sleep quality and sleep latency. Various aspects of mobile phone use such as problem mobile phone use are associated with sleep quality^24^. Presence of significant positive correlations was illustrated between smartphone addiction with each subjective sleep quality, sleep disturbance, daytime dysfunction, and PSQI global scores^2^. Furthermore, Switzerland showed an association between smartphone usage and later bedtimes, but no association with sleep disturbance ^25^. However, a study conducted from Taiwan found no association between MP usage and sleep duration^26^.

The application of our results can be done through building of educational campaigns on dangerous the dangerous of MP overuse. This can decrease the problems. Such campaigns can be done among medical, non-medical or other students.

The limitations and potential biases of the stud was the bias of the cross-sectional study as temporal ambiguity.

## Conclusion

A high frequency and an extensive duration of daily MP usage noticed among medical students from KAU in the current study. The most frequently used smartphone application was What’s App. Being a female, owners of smartphone for more than 1 year, and spending more time on mobile per day were significantly associated with MP dependency. Students with lower academic achievement had significantly worse scores on many of MP addictions domains; namely the financial problems, dangerous MP usage, and with the total PMPU score compared to the higher achievers.‏ Students suffered from poor sleep quality represented about three-fourths of the participants. MP dependency was significantly associated with poor sleep quality as measured by subjective sleep quality and a long sleep latency. Conduction of educational programs about the dangers of increased MP usage is needed for reducing MP dependency. More studies are needed to be done on other physiological and psychological effects of MP usage.

## Acknowledgements

We sincerely thank all students participated in the study and in data collection. We would like to thank also all managers who facilitated its conduction.

## Conflict of interest statement

The authors declare that there is no conflict of interests.

## Funding

No fund.

## Highlights

- Females, owners of smartphone for longer duration & who spent more time on it had more mobile dependency.
- Lower academic achievers had worse score on total Problematic Mobile Phone Use Questionnaire.
- Each of subjective sleep quality score & sleep latency were associated with mobile phone dependency.
- Global Pittsburgh Sleep Quality Index scale was correlated to prohibited mobile phone use.
- Rationale smartphone use is needed for reducing dependency and improving academic achievement.
